# Differentiating anxiety forms and their role in academic performance from primary to secondary school

**DOI:** 10.1371/journal.pone.0174418

**Published:** 2017-03-28

**Authors:** Emma Carey, Amy Devine, Francesca Hill, Dénes Szűcs

**Affiliations:** Centre for Neuroscience in Education, Department of Psychology, University of Cambridge, Cambridge, Cambridgeshire, United Kingdom; University of Pécs Medical School, HUNGARY

## Abstract

**Introduction:**

Individuals with high levels of mathematics anxiety are more likely to have other forms of anxiety, such as general anxiety and test anxiety, and tend to have some math performance decrement compared to those with low math anxiety. However, it is unclear how the anxiety forms cluster in individuals, or how the presence of other anxiety forms influences the relationship between math anxiety and math performance.

**Method:**

We measured math anxiety, test anxiety, general anxiety and mathematics and reading performance in 1720 UK students (year 4, aged 8–9, and years 7 and 8, aged 11–13). We conducted latent profile analysis of students’ anxiety scores in order to examine the developmental change in anxiety profiles, the demographics of each anxiety profile and the relationship between profiles and academic performance.

**Results:**

Anxiety profiles appeared to change in specificity between the two age groups studied. Only in the older students did clusters emerge with specifically elevated general anxiety or academic anxiety (test and math anxiety). Our findings suggest that boys are slightly more likely than girls to have elevated academic anxieties relative to their general anxiety. Year 7/8 students with specifically academic anxiety show lower academic performance than those who also have elevated general anxiety.

**Conclusions:**

There may be a developmental change in the specificity of anxiety and gender seems to play a strong role in determining one’s anxiety profile. The anxiety profiles present in our year 7/8 sample, and their relationships with math performance, suggest a bidirectional relationship between math anxiety and math performance.

## Introduction

Mathematics anxiety (MA) encompasses emotions of fear, tension and discomfort which are felt by some individuals in situations involving mathematics, and which may interfere with one’s performance of mathematical tasks [1]. MA has been seen to relate to math performance in children as young as 5–7 years old [2–4], and this relationship remains in adolescence and adulthood, with two meta-analyses showing correlations of -0.27 and -0.34 between MA and math performance [5,6]. It seems most likely that this relationship is bidirectional, with poor performance contributing to some cases of MA, and MA causing a performance decrement in at least some affected individuals (see [7] for review). Our analysis here aims to investigate how MA is related to other forms of anxiety in a very large sample of 1720 UK children (aged 8–9 years) and adolescents (aged 11–13 years), and how each individual’s anxiety “profile” relates to their academic performance. Measurement of test anxiety and general anxiety as well as MA is novel in a very large sample study, spanning two age groups. Furthermore, we use a combined person- and variable-centered analysis (latent profile analysis), which uniquely enables us to investigate the complex relationship between anxiety forms and performance.

### Math anxiety, test anxiety and general anxiety

Whereas MA is defined as anxiety felt about situations involving mathematics, test anxiety refers to anxiety felt in or about evaluative settings [8]. Test anxiety has long been found to have a negative relationship with test performance, which some have attributed to the idea that test anxiety divides attention between self-relevant variables (such as anxiety-related cognitions) and task-relevant variables, which are required for good task performance [9]. This is comparable to the idea that MA causes a performance decrement by interfering with the working-memory resources required to perform well in some mathematical tasks [1]. As well as being theoretically related, with similar explanations being given for performance decrements in those with test anxiety and MA, the two anxiety types have repeatedly been found to co-occur in individuals, with studies typically reporting moderate correlations between the two constructs [5,10]. This may suggest that MA and test anxiety have some shared “risk factors”–such as a generally anxious personality, teasing about academic performance, or a history of academic difficulties.

General anxiety refers to an individual’s tendency to feel anxious about everyday situations, and tends to involve assessment of areas such as physiological anxiety, worry and social anxiety (these three factors are measured in the Revised Children’s Manifest Anxiety Scale [11]). This construct has a small but consistent relationship with MA [5]. General anxiety might play a role in the relationship between MA and math performance: for example, Hill, Mammarella, Devine, Caviola, Passolunghi & Szűcs [12] found considerable shared variance between MA and general anxiety (e.g. partialling out general anxiety reduced the significant negative relationships between MA and math performance in multiple age groups). General anxiety tends to be less related to MA than test anxiety [5]. This is coherent with the idea that the relationship between anxiety forms relates to shared risk factors: test anxiety and MA are likely to have more similar risk factors (e.g. those which root in experiences of school and achievement) than general anxiety and MA.

The relationship between test anxiety, general anxiety and MA gives strong rationale to measure all three variables in order to investigate the presence of subgroups of students with different forms of anxiety. This should provide great insight into developmental change in anxiety forms, enable further conclusions on the mechanisms of the relationship between MA and performance and inform research into interventions for students with different profiles of MA and other anxiety forms.

### The use of latent profile analysis to identify anxiety subgroups

Whilst each anxiety construct appears to be related to the others, it appears that each anxiety type also has a large proportion of variance which is *not* reflected in other anxiety types. Hembree [5] reports that after adjusting for attenuations based on instrument reliability, the corresponding coefficient of determination between MA and test anxiety was r^2^ = 0.37. In other words, 37% of variation in MA can be accounted for using test anxiety. The fact that general anxiety and MA are consistently less strongly correlated than are test anxiety and MA means that even less variation in MA can be accounted for by general anxiety. The factors discussed as accounting for some of the covariation between anxiety types are likely to interact with other personal and environmental characteristics in a complex fashion, meaning that some individuals have disproportionately high levels of one or two anxiety types over the other(s).

Since none of the anxiety types appear to be entirely subsumed by any other, it is important to examine how these distinct but related forms of anxiety appear within certain individuals. The majority of research into MA has utilized variable-centered approaches such as correlations and regression analysis. These approaches give some idea of the overall relationships between variables, but cannot elucidate the presence of heterogeneous subgroups within a population. An integrated person- and variable-centered analysis can help identify the presence of such subgroups, and explain why classical variable-centered analyses account for only a small proportion of variance in MA between individuals [13].

Latent Profile Analysis (LPA) is one such integration of person- and variable-centered analyses. LPA is similar to Latent Class Analysis (LCA), but whereas LCA is confined to analyses of categorical variables, LPA can be used in situations where the variables measured are continuous, or a mixture of categorical and continuous. Based on the levels of the variables which have been measured, LPA classifies individuals from a large, heterogeneous population into smaller, more homogenous subgroups. This is based on the assumption that an individual’s level of the measured variables depends on categorical, latent (unmeasured) variables [14]. LPA is a form of mixture model, based on the idea that data are not sampled from a population of one probability distribution but from one with a mixture of separate distributions (one at each level of the categorical latent variable) [15].

The fact that MA’s relationship with the other anxiety forms is poorly captured by simple, variable-focused analyses suggests that there may be some use in applying a latent profile mixture model to classify different profiles of anxiety which occur within a population. This could identify subgroups of individuals who express certain anxiety patterns, rather than imagining that test anxiety, general anxiety and MA are similarly related to one another in every individual, as purely variable-centered analyses do.

### Hypotheses regarding anxiety profiles and their performance outcomes

Firstly, one would expect the identification of a large, “normative” profile with below average scores on each anxiety measure, due to having few risk factors for any of the anxiety types. One would expect this group to primarily consist of boys, who tend to have lower levels of general anxiety [16], test anxiety [17] and MA [5]. We predict that such a group would have above average performance in math, since they are free of anxieties which would cause a performance decrement. There is no reason to believe that the reading performance of this group would be anything other than intact.

A previous study in younger children suggests that, in 9-year-olds, LPA subgroups tend to reflect high, medium or low anxiety *in general* [18]. However, there is no such research available for older children. We hypothesize that, particularly in older children, some individuals may be affected greatly by factors which increase their propensity to anxiety in general, such as female gender [5,16,17]. These individuals would be likely to express high levels of all forms of anxiety, including general anxiety, test anxiety and MA. We would expect this group to have below average math performance, since, regardless of MA’s etiology, it appears to affect working-memory and thus the solving of math problems. However, this group are likely to have developed MA as a result of a general disposition to anxiety, rather than because they have poor self-confidence in math specifically. Therefore, their performance in math may be normal if not for the interfering effect of MA, and they may have less of a math performance deficit than groups who either have specific math or academic anxiety (that is, MA and TA).

Other individuals might be free of factors which encourage general anxiety to form, but have specific risk factors for academic anxiety. It is likely that some children have specifically high academic anxiety (encompassing test anxiety and MA), since academic self-perceptions are more correlated with each other than they are with self-esteem generally (r = 0.64 between self-perceptions of math competence and school competence in [19]). Compared with the group who are high in all anxiety forms, this group are less likely to have a strong general predisposition to anxiety (in which case one would expect their general anxiety levels to be high as well as their academic anxiety). Thus this group are more likely to have developed academic anxiety as a result of poor academic self-perception, which may emerge as a result of experiences with poor past performance. Therefore, we expect that a group experiencing specific academic anxiety will have poorer math performance than a group with high anxiety in general, since they are more likely to have had poor performance prior to the development of their anxiety.

## Materials and methods

### Sample

1849 students were tested, at primary and secondary schools across Cambridgeshire (8 schools), Suffolk (7 schools), Hertfordshire (7 schools), Norfolk (2 schools) and Bedfordshire (1 school). Students were recruited and tested in 2014. 1720 students remained after deleting those with more than one item missing from their anxiety questionnaires. For those 173 students with only one item missing, the item was replaced by the participant’s average item score for that questionnaire. The year 4 sample consisted of 817 students (mean age = 109.4 months, SD = 3.7 months), 402 girls and 415 boys. The year 7/8 sample consisted of 903 students (mean age = 148.0 months, SD = 4.0), 391 girls and 426 boys.

School demographics varied widely, with locations being both urban and rural. A school’s percentage of students receiving Free School Meals (FSM) can be used as an indicator of SES, since consistent economic criteria are used nationwide to determine a child’s entitlement to FSM [20]. Schools in this sample varied from 2.9% to 36.5% receiving FSM [21], with schools falling both above and below the national average (calculated as 20.9% of 11 year olds in 2014, from figures in [22]). Schools also varied widely in the percentage of students with special educational needs (SEN) and who had English as an additional language (EAL). Students were not excluded on the basis of SEN or EAL, in order to increase the representativeness of the sample.

Ethical permission was obtained from the Psychology Research Ethics Committee of the University of Cambridge, and opt-out consent was used. Each child brought home a slip to return if the parents did not wish them to participate in the study. Opt-out parental consent was considered acceptable for this study, since the questionnaires and tests taken by the students were within the realms of what can be expected as part of the school day. We were sensitive to distress displayed by any child, who would have been allowed to withdraw without penalty.

### Materials

#### General anxiety

The Short Form of the *Revised Children’s Manifest Anxiety Scale*: *Second Edition* [11] was used to measure general anxiety. Because of time constraints when screening such a large sample, the 10-item Short Form was used in preference of the full 49-item questionnaire. Participants answer in a yes/no format, resulting in a score from 0–10. The Short Form RCMAS-2 has been shown to be adequately reliable [11]. Cronbach α was 0.74 (95% confidence interval 0.71–0.76) in the current sample, suggesting adequate reliability.

#### Test anxiety

The Children’s Test Anxiety Scale [17] was used to assess test anxiety. The CTAS assesses children’s reactions in various testing situations using self-report on 30 items. These items assess a child’s thoughts (e.g. “I think about what will happen if I fail”), off-task behaviors (e.g. “I play with my pencil”) and autonomic reactions (e.g. “My hand shakes”). Participants respond on a four-point Likert scale (from “almost never” to “almost always”) resulting in scores from 30–120. The CTAS has been shown to have adequate reliability (α = 0.92) and construct validity [17]. In the current sample, Cronbach α was 0.92 (95% confidence interval 0.91–0.93), suggesting excellent reliability.

#### Mathematics anxiety

MA was measured using a modified version of the Abbreviated Math Anxiety Scale [23]. The AMAS is a 9-item self-report questionnaire, in which participants use a 5-point Likert scale to indicate how anxious certain math situations would make them feel, with scores ranging from 9–45. Research indicates that this short scale is as effective as the longer Math Anxiety Rating Scale [23], having adequate internal consistency (Cronbach’s α = 0.90), two week test-retest reliability (r = 0.85) and convergent validity with the MARS-R (r = 0.85). Participants rate how anxious they would feel in certain situations involving math using a 5-point Likert scale, with a maximum score of 45.

The modified AMAS (mAMAS) has been used previously with a sample of British primary school children [24]. The modifications to the AMAS involved minor adjustments to convert US English to British English. Additionally, items which refer to advanced math (e.g. “Checking the tables in the back of a textbook”) were altered to increase appropriateness for a primary sample (e.g. to “Completing a worksheet by yourself”). Cronbach’s alpha for the mAMAS in the current study was 0.85 (95% confidence interval 0.83–0.87), suggesting that the mAMAS has good internal consistency. Furthermore, we have shown through factor analysis that the mAMAS measures a unique construct, MA, independent from general and test anxiety [25].

#### Mathematics performance

The Mathematics Assessment for Learning and Teaching (MaLT [26]) was used to assess children’s math performance. These tests were developed in accordance with the National Curriculum and National Numeracy Strategy for England and Wales. Students were given the appropriate MaLT test for their level of schooling: year 4 students took the MaLT 9, year 7 students took the MaLT 12 and year 8 students took the MaLT 13. These tests covered age-appropriate mathematics content, such as counting and understanding number, use and knowledge of number facts, calculations, understanding of shape, measurement and data handling. Students had 45 minutes to complete the relevant tests. The tests were standardized using a sample of 12,591 children from 120 English and Welsh schools [27], and all show strong internal consistency (MaLT 9: α = 0.93, MaLT 12: α = 0.92, MaLT 13: α = 0.93).

#### Reading performance

Hodder Group Reading Tests II (HGRT-II [28]) were used to assess the children’s reading performance. These tests include multi-choice and free choice items which test children’s understanding of words, sentences and passages. Two parallel versions of the test were used for each age group, to discourage students from copying from nearby children. Children were given the appropriate HGRT-II for their schooling level: year 4 students took the HGRT-II level 2 and year 7 and 8 students completed HGRT-II level 3, and each test took 30 minutes. The children had 30 minutes to complete the relevant test. The HGRT-II was standardized in 2005 on a large sample of more than 13,000 pupils from 111 English and Welsh schools, and both relevant tests show strong internal consistency (HGRT level 2: α = 0.95, HGRT level 3: α = 0.94).

### Procedure

Tests and questionnaires were administered by researchers in the children’s schools. These assessments took place in sessions of around 2 hours, in groups of 25–150. The order of tests and questionnaires was counterbalanced between schools, to avoid any effects on anxiety levels created by having just taken a test, or on test scores having just taken an anxiety assessment.

Care was taken to present the testing material to the year 4 school students in an accessible, ‘child-friendly’ manner. A PowerPoint slide-show was given to explain the tasks and the meanings of less common words (e.g. ‘anxiety’ was defined in terms of feeling nervous, worried or scared). All questionnaire items were read aloud to help those students with reading difficulties, and practice items were included to ensure the children’s understanding. Questionnaire items were re-formatted to be presented in a readable booklet and happy and sad faces were added to the relevant ends of the AMAS and CTAS Likert scales, to aid students with their responses.

### Data analysis

For ease of display, each anxiety score was placed on a 0–1 scale. This was done by subtracting the minimum possible score for that scale from the participant’s actual score, and dividing the outcome by the maximum possible score on the scale. Initially, Spearman’s correlation coefficients between scaled anxiety and standardized performance measures were examined, to investigate relationships between all anxiety measures and both performance measures. Because of age-related differences in relationships between anxiety and performance measures, it was decided that the two discrete age groups should be examined separately.

LPA is an empirical method used to define subgroups of people with similar characteristics from a more heterogeneous population. Using maximum likelihood estimation [29], LPA defines a set of classes based on all observations of the continuous dependent variables in question (e.g. scores on each of the three anxiety measures). As the program generates a structure of profiles, it places each individual into their most likely profile and estimates the likelihood that each individual has been placed into the correct category. LPA is used to generate models with classes added iteratively, and both substantive knowledge and model fit are used to determine the optimal number of classes.

Mplus v.7.11 [30] was used to conduct LPA for this study. Variance was constrained to be equal across profiles, since there was no rationale to change this default setting. Models were evaluated using the Lo-Mendell-Rubin Adjusted Likelihood Ratio Test (LMR LRT) [31], Bootstrapped Likelihood Ratio Test (BLRT) [32]. These tests indicate whether the number of profiles in a given model is enough of an improvement on the model with one fewer profiles to justify the additional profile (p < 0.05 if the model fit is a significant improvement). Akaike Information Criterion (AIC) [33] and Bayesian Information Criterion (BIC) [34] were also used to provide descriptive indices of each model’s fit, and informed our model choice. Classes in each model were examined to ensure that no spurious classes had emerged (a possible consequence of extracting too many profiles from the data [35]). Where statistics were in conflict, we chose to use the principle of parsimony and tended to choose the simpler of available models in order to avoid reaching local solutions (the importance of parsimony is discussed in [36]).

After running LPA, conducting substantive interpretation of the best fitting solutions, and naming profiles accordingly, we ran linear regression models predicting mathematics performance based on MA. LPA profile was then added to the linear regression models in order to gauge whether LPA profile had an impact on mathematics performance above and beyond the effect of MA levels in each profile.

## Results

### Descriptive statistics and correlations

**Fig 1:**
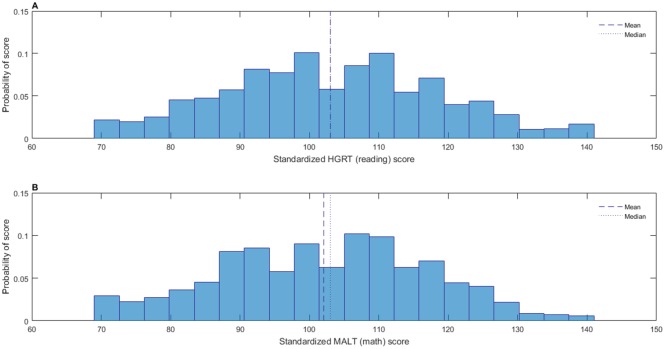
Performance score distributions. Distributions of (A) standardized reading performance scores and (B) standardized math performance scores.

**Fig 2:**
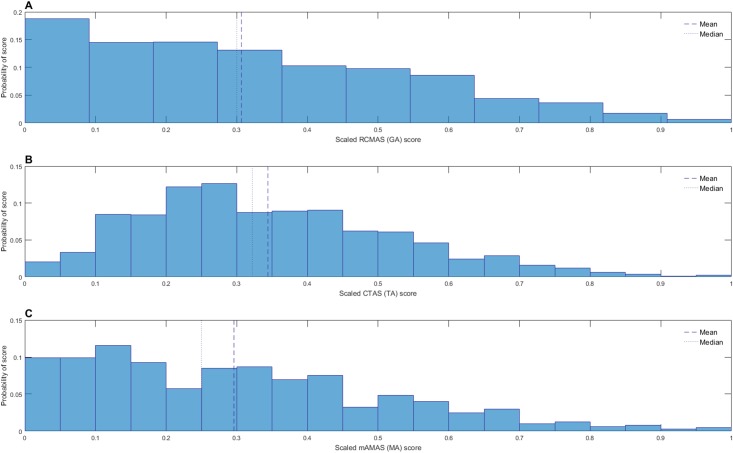
Anxiety score distributions. Distributions of (A) scaled general anxiety (GA) scores, (B) scaled test anxiety (TA) scores and (C) scaled MA scores.

Prior to analysis, HGRT (reading performance) and MaLT (math performance) scores were standardized according to published norms (see section *2*.*2*.*4* and *2*.*2*.*5* for more information). General anxiety, test anxiety and MA scores were scaled as described in section *2*.*4*). Fig 1 shows distributions of standardized performance scores and Fig 2 shows distributions of scaled anxiety scores. Table 1 shows descriptive statistics for unscaled and scaled anxiety scores and standardized and unstandardized performance scores and Spearman’s rho between each standardized/scaled measure.

**Table 1:** Performance and anxiety scores and correlations.

		Year 4					Year 7/8				
		HGRT	MaLT	GA	TA	MA	HGRT	MaLT	GA	TA	MA
**Raw**	**Mean**	34.92	23.05	3.36	60.51	19.26	30.81	20.08	2.80	61.36	20.00
	**SD**	10.78	9.94	2.47	17.46	7.84	9.53	10.04	2.49	15.21	7.52
**Standardized/ scaled scores**	**Mean**	105.63	103.23	0.34	0.34	0.29	100.64	100.99	0.28	0.35	0.31
	**SD**	16.02	15.53	0.25	0.19	0.22	14.81	14.54	0.25	0.17	0.21
**Correlations and *p* values**	**HGRT**		0.74	-0.16	-0.14	-0.14		0.70	0.01	-0.09	-0.17
	**MaLT**	0.699, 0.767		-0.27	-0.26	-0.31	0.660, 0.729		-0.09	-0.16	-0.28
	**GA**	-0.225, -0.094	-0.332, -0.205		0.63	0.50	-0.053, 0.077	-0.156, -0.023		0.60	0.53
	**TA**	-0.210, -0.073	-0.325, -0.195	0.579, 0.700		0.71	-0.155, -0.024	-0.222, -0.094	0.548, 0.641		0.69
	**MA**	-0.210, -0.072	-0.370, -0.241	0.443,0.558	0.666, 0.750		-0.234, -0.107	-0.340, -0.217	0.479, 0.581	0.650,0.728	

Raw and standardized reading (HGRT) and math (MaLT) performance scores and raw and scaled general anxiety (GA), test anxiety (TA) and math anxiety (MA) scores, and Spearman’s correlation coefficients and bootstrapped 95% confidence intervals (calculated with 10,000 permutations using MATLAB).

*Note*. Spearman’s rho is shown in the cells above the diagonal line of blank cells, and bootstrapped 95% confidence intervals below this line.

In both age groups, MA was correlated with both math and reading performance. Whilst these correlations are significant, they are not particularly strong, indicating that MA only explains a small portion of the overall variation in math performance, and even less of the variation in reading performance. The fact that general anxiety was only linked to math and reading performance in year 4 students gave sufficient evidence to analyze each age group’s data separately, in case the relationships between anxiety and performance differ by age.

**Fig 3:**
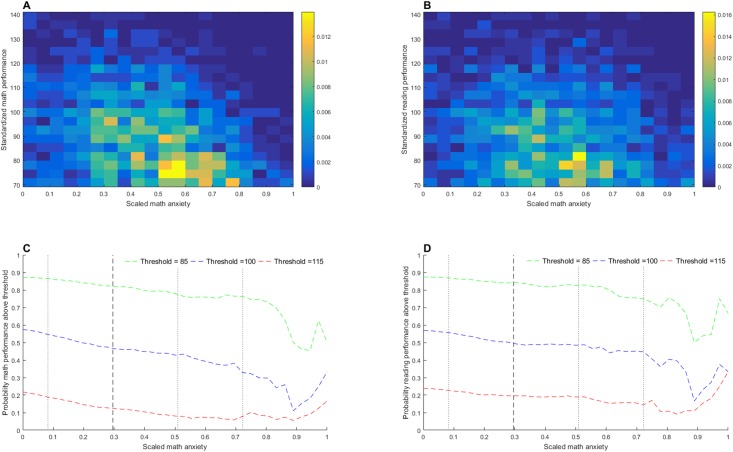
The Relationship between academic performance and math anxiety. (A) Density scatter plot showing probability of each standardized math performance score at each scaled MA level. (B) Density scatter plot showing probability of each standardized reading performance score at each scaled MA level. (C) Conditional probability of standardized math performance being equal to or above the specified threshold at each scaled MA level. (D) Conditional probability of standardized reading performance being at or above the specified threshold at each scaled MA level.

Fig 3 shows density scatter plots and diagrams of conditional probabilities for the relationships between MA and both math and reading performance. These graphs demonstrate that whilst math and reading performance are both related to MA, individuals with the same MA score have highly variable performance. Despite the consistent relationship between MA and performance, even those with MA levels more than 1 standard deviation above the mean have a reasonable chance of performing better than average in math and reading.

### Latent profile analysis of anxiety types

LPA of the anxiety scores was run separately for younger and older children, to see how the profiles discovered related to math and reading performance. LPA was carried out using scaled anxiety scores. All fit statistics are reported in Table 2. For the year 4 students, based on a Lo-Mendell-Rubin adjusted Likelihood Ratio Test (LRT), a 4-profile solution was optimal. On the other hand, the BLRT continued to suggest that models with a greater number of profiles were significantly superior. AIC, BIC and entropy did decrease and increase respectively between a 4-profile and 5-profile solution; however, the BIC difference of 5 between a 4-class and 5-class model does not suggest that parsimony should be sacrificed to accept the 5-class model. Furthermore, one class in the 5-class model contained only 6 participants (0.007 of the entire sample); thus this class was not examined further and the 4-class model was accepted.

**Table 2:** Measures of LPA model fit.

		AIC	BIC	LRT Value	LMR LRT p-value	BLRT p-value	Entropy	Substantive examination
**Year 4**	**2-profile**	-1258	-1201	780.11	<0.001	<0.001	0.804	OK
	**3-profile**	-1457	-1373	211.08	0.008	<0.001	0.757	OK
	***4-profile***	***-1535***	***-1422***	***89*.*31***	***0*.*004***	***<0*.*001***	***0*.*774***	***OK***
	**5-profile**	-1569	-1427	99.68	0.59	<0.001	0.805	Smallest class <0.01
**Year 7/8**	**2-profile**	-1803	-1746	963.14	<0.001	<0.001	0.845	OK
	**3-profile**	-1994	-1907	913.60	<0.001	<0.001	0.743	OK
	***4-profile***	***-2105***	***-1990***	***123*.*42***	***<0*.*001***	***<0*.*001***	***0*.*777***	***OK***
	**5-profile**	-2161	-2017	66.38	0.15	<0.001	0.788	Unnecessarily splits class

Akaike Information Criterion (AIC), Bayesian Information Criterion (BIC), Lo-Mendell-Rubin adjusted Likelihood Ratio Test (LMR LRT), Bootstrapped Likelihood Ratio Test (BLRT), Entropy values and results of substantive examination for solutions with the given number of profiles.

*Note*. p-values indicate whether or not the LRT test suggests the model with n classes is significantly better than the model with n-1 classes. Emboldened, italicized rows indicate our chosen model for each age group, considering AIC, BIC, entropy and likelihood-ratio tests.

For year 7/8 students, the LRT also showed a 4-profile solution to be optimal. Again, the BLRT suggested that a more complex model may be preferable. However, decreases in AIC, BIC and increases in entropy began to flatten when the number of profiles was increased from 4 to 5. Furthermore, the difference between the 4 and 5-profile models merely involved a splitting of the highest anxiety profile (into two profiles with fairly homogenous scores on each form of anxiety). This was considered an unnecessary complexity, and thus the more parsimonious 4-profile model was accepted for further analysis.

**Fig 4:**
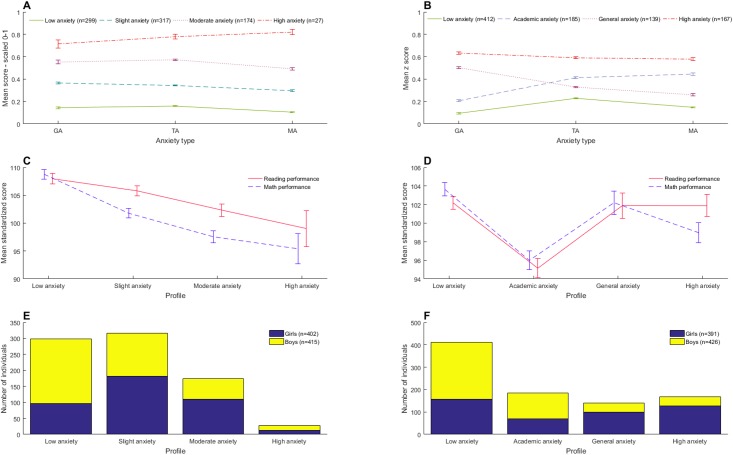
Anxiety, gender and performance in each LPA profile. (A) Line graph showing mean levels of general anxiety (GA), test anxiety (TA) and math anxiety (MA) in each LPA profile for year 4 children. (B) Line graph showing mean levels of GA, TA and MA in each LPA profile for year 7/8 children. (C) Stacked bar graph showing the number of girls and boys in each LPA profile for year 4 children. (D) Stacked bar graph showing the number of girls and boys in each LPA profile for year 7/8 children. (E) Line graph showing mean standardized reading and math performance score in each LPA profile for year 4 children. (F) Line graph showing mean standardized math and reading performance in each LPA profile for year 7/8 children. All error bars show standard error of the mean.

Fig 4A and 4B shows the mean anxiety score of each profile for each age group. Each profile for the year 4 students exhibits fairly homogenous mean scores for each anxiety measure. On the other hand, the year 7/8 students’ profiles have more varied means for each anxiety measure, with one profile having a lower mean general anxiety but higher test and MA than another. This indicates that more varied and topic-specific anxiety types were present for a larger subset of individuals in the year 7/8 sample, whereas in the year 4 sample the majority of individuals either had high, medium or low anxiety *in general*.

The profiles have been named descriptively for ease of identification. Profile names in the year 4 sample are “High anxiety”–the group with highest average scores on all three anxiety measures; “Moderate anxiety”–the group with medium-high scores on each anxiety measure; “Slight anxiety”–the group with medium-low scores on each measure and “Low anxiety”–the group with the lowest average scores on all anxiety measures. The year 7/8 sample also consists of a “Low anxiety” and “High anxiety” group, which are lower and higher respectively than all other profiles in this age group on every anxiety measure. The two medium anxiety groups were named “General anxiety”–the group with higher general but lower test anxiety and MA, and “Academic anxiety”–the group with higher math and test anxiety but lower general anxiety. These descriptive names are used in Fig 4 and henceforth.

### Latent profile membership and gender

Gender appears to influence an individual’s likelihood of being in each LPA profile. Chi-square tests of independence were calculated comparing the frequency of profile membership in girls and boys. In year 4 children this indicated that there was a significant interaction between gender and LPA profile (χ^2^(3) = 55.85, *p* < 0.001). Girls were more likely to be in the higher anxiety profiles and boys in the “Low anxiety” profile (see Fig 4E for graphical representation). There was also a significant interaction between gender and LPA profile in year 7/8 children (χ^2^(3) = 110.40, *p* < 0.001). Girls were more likely to be in the “General anxiety” and “High anxiety” profiles, and boys in the “Low anxiety” and “Academic anxiety” profiles (see Fig 4F for graphical representation).

### Latent profile membership and performance

Fig 4C and 4D shows mean math and reading performance for each latent profile, with error bars displaying standard error of the mean. To confirm that math performance varies significantly according to class membership for each age group, one way ANOVAs were conducted for each age group, with standardized math performance as the dependent variable and latent profile membership as the independent grouping variable. One-way ANOVAs showed a significant effect of LPA profile on math performance in year 4s (*F*(3,813) = 25.80, p < 0.001) and year 7/8s (*F*(3,899) = 13.82, *p* < 0.001). LPA profile also had a significant effect on reading performance in year 4s (*F*(3,813) = 6.22, *p* < 0.001) and year 7/8s (*F*(3,899) = 11.00, *p* < 0.001).

These results confirm the expected outcome that latent profile membership is predictive of mathematics and reading performance. However, they give no indication of whether this predictive ability exists once MA scores are accounted for: i.e. the relationship between latent profile membership and performance could be entirely driven by the different levels of MA in each profile.

In year 4 school students, the relationship between LPA profile and anxiety scores is rather simplistic. Higher levels of anxiety in all forms result in a higher anxiety LPA profile. On the other hand, in year 7/8 students, LPA profile reveals something about the pattern of anxieties a student experiences, above and beyond their maths anxiety score. Thus we wished to investigate whether, in year 7/8 students, performance can be predicted more accurately when information about a student’s LPA profile is accounted for.

In order to assess whether LPA profile enables better prediction of math and reading performance when compared with MA level alone, we ran linear regression models predicting math and reading performance from MA (for year 7/8 students). We then ran these models again with gender as an additional predictor of math and reading performance. Using a likelihood ratio test, we selected the optimal model. We then added LPA profile to this, to see whether this would significantly improve the predictive power of the model. Table 3 shows model fit for math and reading performance in year 7/8 students. Table 4 shows coefficients for each independent variable in the optimal model for each dependent variable in each age group. LPA profile significantly added to models predicting math and reading performance from MA score (and gender, in the case of reading) in year 7/8 age students. As can be seen from the beta coefficients in Table 4, this is because individuals with high scores in all anxiety forms (the “High anxiety” profile) did relatively better than those in other profiles, and in particular compared with those in the “Academic anxiety” profile. This can also be seen in Fig 4D.

**Table 3:** Regression model fit statistics for secondary students.

Dependent variable	Independent variables	Model fit statistics				Model comparison		
		*R*^2^	AIC	BIC	Log-likelihood	Compared with	LR stat	*p* value
**Math performance**	MA		7327	7341	-3660			
	MA + gender		7329	7348	-3660	Model 1	0.03	0.86
	***MA + LPA profile***		***7311***	***7340***	***-3650***	***Model 1***	***21*.*56***	***<0*.*001***
**Reading performance**	MA		7409	7424	-3702			
	MA + gender		7392	7411	-3692	Model 1	19.65	<0.001
	***MA + gender + LPA profile***		***7371***	***7405***	***-3679***	***Model 2***	***46*.*12***	***<0*.*001***

Each regression model’s dependent and independent variables, their fit statistics, and statistics from a likelihood ratio test (LRT) used to compare models.

*Note*. Model 1 predicts performance in either maths or reading from MA alone. Model 2 predicts performance based on MA and gender. An LRT is used to compare this to the more basic model. If model 2 is preferable to model 1 (p<0.05), model 3 is formed by adding LPA profile as an additional predictor to model 2. If model 2 is not preferable to model 1, model 3 is formed by adding LPA profile to model 1. For each dependent variable at each age, the optimal regression model is emboldened and italicized.

**Table 4:** Optimal regression model statistics for secondary students.

Dependent variable	Independent variables	Coefficients			
		Beta	SE	*t*	*p* value
**Math performance**	***MA + LPA profile***				
	Intercept	108.01	0.89	121.84	<0.001
	MA	-29.44	3.85	-7.65	<0.001
	LPA profile—Academic anxiety	1.08	1.67	0.65	0.52
	LPA profile—General anxiety	1.83	1.41	1.29	0.20
	LPA profile—High anxiety	8.00	2.08	3.84	<0.001
**Reading performance**	***MA + gender + LPA profile***				
	Intercept	107.49	1.12	96.08	<0.001
	MA	-22.48	3.98	-5.65	<0.001
	LPA profile—Academic anxiety	-0.33	1.73	-0.19	0.85
	LPA profile—General anxiety	1.13	1.50	0.75	0.45
	LPA profile—High anxiety	8.15	2.18	3.74	<0.001
	Gender—Male	-3.20	1.01	-3.16	0.002

Dependent and independent variables, estimated beta coefficient (Beta), standard error (SE), t-statistic (t) and p-value for the optimal regression model predicting math and reading performance for each age group.

## Discussion

### Correlations

The correlation of -0.29 found in our large sample between MA and math performance reflects that observed in meta-analyses [5,6], and suggests that whilst MA is linked with poorer math performance, there are many further factors which determine a child’s performance outcome in math. The fact that there is a correlation of -0.17 between MA and reading performance could result from children who develop MA as a result of poor math performance, because math and reading performance are closely related (in this sample, there was a correlation of 0.73 between math and reading performance).

Alternatively, it might be the case that those children with MA are more likely to have other forms of academic anxiety which impact their reading performance. Some researchers have proposed the construct of literacy anxiety and, if this is related to MA, individuals with MA might be more likely to have poor reading performance because of co-existing literacy anxiety (e.g. [18]) suggest that those with literacy worries tend to also have worries about math and have reduced reading performance). Because we did not measure reading anxiety in our participants it is impossible to judge whether one or both of these mechanisms operates to explain the correlation between MA and reading performance.

### Latent profile analysis

LPA run using students’ scores on each of the three anxiety measures found different anxiety “profiles” amongst year 4 students than amongst year 7 and 8 students. LPA of year 4 students’ anxiety scores yielded four profiles. One group had low scores on all anxiety measures (“Low anxiety”), another had low-medium scores on all anxiety measures (“Slight anxiety”), another had medium-high scores on all anxiety measures (“Moderate anxiety”) and the last had high scores on all anxiety measures (“High anxiety”). This accords with Punaro & Reeve’s [18] data, suggesting that grouping young children on the basis of their anxiety scores does not yield groups with differing patterns of scores on each measure.

On the other hand, LPA of year 7 and 8 students’ scores on each anxiety measure yielded four groups with differing patterns of scores on each of the three anxiety types. One group, as predicted, consisted of students with normative scores on all three measures and was thus named “Low anxiety”. A second group (“General anxiety”) showed high scores only on the measure of general anxiety. A third group (“Academic anxiety”) showed a pattern of high scores on test anxiety and MA, our two anxiety measures concerned with academia. The final group (“High anxiety”) had high scores on all three anxiety measures. The presence of these more specific anxiety clusters in year 7/8 students enabled us to test our hypotheses regarding the performance outcomes of children in each profile.

There is no way to test whether the difference in profiles identified between year 4 and year 7/8 reflects a significant developmental change. However, it raises the interesting possibility that variance in MA in younger children could be primarily driven by a general tendency towards anxiety, whereas that in older children may reflect more specific anxiety forms. Further testing to see whether this pattern of results can be replicated may help to inform as to whether this shows a genuine developmental change in anxiety specificity. Further evidence supporting a developmental change in anxiety specificity can be seen in Table 1. The confidence interval for *r* between math performance and general anxiety in year 4 children does not overlap with the confidence interval for *r* between the same variables in year 7/8 children. The same holds for the confidence interval for *r* between reading performance and general anxiety.

If such a developmental change exists, longitudinal research into how the anxiety forms present in year 4 students are precursors to those in year 7/8 students might enable the development of early interventions for students with high anxiety. It may also enable further explanations for other research findings. For example, Hill et al. [12] found that the relationship between MA and performance remained significant in secondary but not primary students after partialling out general anxiety. This could be explained by the differential clustering of students with MA and general anxiety in year 7/8 compared with year 4.

### The Relationship between anxiety profile and gender

Gender appears to strongly affect an individual’s likelihood of belonging to each LPA profile, at both age groups tested. Prior research consistently implicates female gender in risk for MA, test anxiety and general anxiety [5,16,17]. Thus it is unsurprising that being female raised one’s likelihood of being in higher anxiety profiles and being male raised one’s likelihood of being in lower anxiety profiles. Looking at MA and test anxiety alone, it appears that girls are at more risk of experiencing these academic anxieties than boys. However, our data demonstrate that year 7/8 boys are at higher risk of belonging to the “Academic anxiety” LPA profile than girls. That is, whilst girls have an overall higher level of academic anxiety than boys, boys are more likely to fall into the profile with high test anxiety and MA relative to their general anxiety level.

This might suggest that whilst girls with academic anxieties often develop them because of a general predisposition to anxiety, some boys develop relatively high levels of academic anxiety without having a predisposition towards general anxiety. Thus typical etiology of anxieties surrounding school might differ between boys and girls. This could be important as regards the type of intervention and support required for individuals of different genders.

### The Relationship between anxiety profile and math and reading performance

#### Year 4 students

Year 4 students’ anxiety profile was related to both math and reading performance. Students in the lowest anxiety profile averaged high performance in both mathematics and reading. Those in the slight anxiety profile showed poorer math performance and slightly poorer reading performance than those in the lowest anxiety profile. Those in the moderate anxiety profile showed more impairment than this. Those in the highest anxiety profile had the lowest scores in math and reading. This pattern of results was confirmed using ANOVA, but is to some degree unsurprising. In year 4 students the relationship between MA and latent profile was very straightforward, and it has been demonstrated many times (e.g. see [5]) that there is a negative relationship between MA and math performance.

#### Year 7/8 students

In year 7/8 students, LPA led to the identification of more varied anxiety profiles. These profiles were found to relate to math and reading performance in a more complex way than could be seen in year 4 students.

We found that including LPA profile in linear regressions predicting math and reading performance from MA led to a significant improvement in the regression models, according to Likelihood Ratio tests. This occurred because children in the “High anxiety” profile had significantly higher math performance than would be predicted by their MA alone, and significantly higher reading performance than would be predicted by their MA and gender (see Table 4). This suggests that whilst those in the “High anxiety” profile do have some impairment in mathematics, this impairment is not as great as it is in children who have specifically high academic anxieties.

Some theories of the MA-performance relationship focus entirely on the deleterious effects of MA, often alongside other anxieties, on performance (e.g. [1]). However, these theories would predict that a group of children who had higher levels both of MA and other anxiety forms would perform *worse* than those with lower levels of all anxiety types. This is not the case: it appears, counter to this theory, that having other forms of anxiety reduces the performance decrement caused by MA. To explain this finding, it is important to consider that MA may have a different etiology in different groups of children, and thus relate differentially to performance in different groups.

The Deficit Theory suggests that MA is elicited by experiences of poor performance in math, and that this explains the relationship between MA and performance (supported by [37]). The Deleterious Anxiety Model, on the other hand, suggests that children with MA have impaired math performance because anxiety interferes with cognitive processes, such as working memory [38] (see [7] for review of these theories). Our findings run counter to predictions of both the Deficit Theory and the Deleterious Anxiety Model alone. If the mechanisms of the Deficit Theory acted alone to drive the relationship between MA and performance, one would not expect a clustering of MA with other forms of anxiety, which suggests that one’s general disposition towards anxiety (not one’s performance) is a major driver of MA.

On the other hand, the Deleterious Anxiety Model would suggest that those with the absolute highest levels of anxiety should perform the worst. The effect of adding LPA profile to a linear regression predicting math performance from MA suggests that the Deleterious Anxiety Model is not the only driver of the relationship between MA and math performance. This shows that, relative to their absolute MA levels, the “High anxiety” profile have higher math performance than other profiles.

To explain these data, we propose that MA can develop via two main mechanisms. Some students (those in the “High anxiety” group) appear to be predisposed towards anxiety in all forms tested, and thus may develop MA regardless of their mathematics experiences and performance. Other students do not seem to have this risk factor towards general anxiety, but do develop relatively high levels of academic anxiety (the “Academic anxiety” group). These students have particularly low mathematics performance, suggesting that as well as MA reducing their math performance, some of them might have had poor math performance because of an additional factor (e.g. lower IQ or working memory) than their MA. This might be what led them to develop relatively high academic anxiety in the first place. This suggestion is bolstered by the fact that students in the “Academic anxiety” group have significantly lower *reading* performance than those in the “High anxiety” group. Math and reading performance are highly related, and there is no suggestion in our data that this occurs because of a mechanism related to anxiety, in which case one would expect the “High anxiety” profile to have the lowest reading performance. Thus there is a likelihood that individuals with a factor reducing their general academic performance, are likely to develop MA (and test anxiety) via the mechanisms of the Deficit Theory, whereas others develop MA because of a predisposition to anxiety generally but without poor academic performance. This group does still develop poorer math performance relative to non-anxious students, likely by the mechanisms of the Deleterious Anxiety Model.

**Fig 5:**
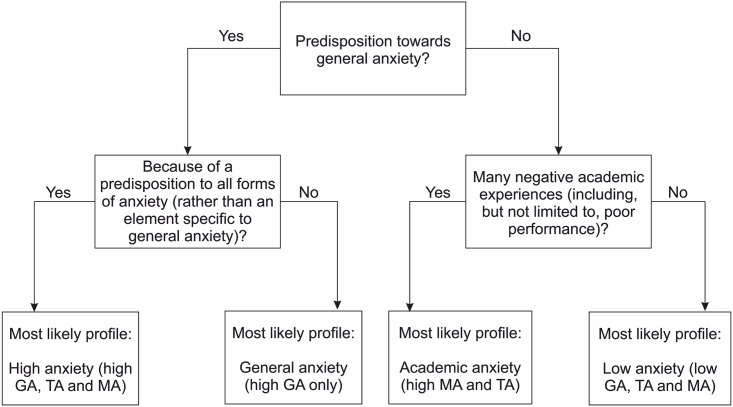
Hypothesized model of LPA profile determination. Simplified binary-choice diagram suggesting factors which might influence a child’s LPA profile via their levels of general anxiety (GA), test anxiety (TA) and MA. Note that children in the “High anxiety” profile are likely to have developed higher MA levels as a result of a general predisposition to anxiety, whereas those in the “Academic anxiety” profile are more likely to have developed high MA as a result of poor academic performance. This explains why those in the “High anxiety” profile have higher academic performance relative to their absolute MA levels than those in the other profiles.

The diagram in Fig 5 demonstrates potential mechanisms for the development of each anxiety profile revealed by LPA. Whilst it is based on binary answers to each question asked, the answers to these questions do not in reality fall into binary categories, and it is likely that various anxiety forms are affected by a matter of degree (rather than categorically) by each of the variables in question. For example, somebody with almost entirely negative academic experiences is more likely to fall into the “Academic anxiety” profile than someone with a mixture of negative and positive experiences. Even within each profile, the absolute levels of anxiety exhibited by each person are highly variable (though less so than within the group as a whole), and it is vital to remember that there are such a huge quantity of possible biological and experiential factors which influence an individual’s personal levels of anxiety.

However, this diagram broadly outlines how year 7/8 school students’ anxiety profiles might form. It is in accordance with our data on the math and reading performance of students in each anxiety profile: all groups of students with high MA (whether or not this comes alongside other forms of anxiety) have some level of mathematical impairment, as would be expected under the Deleterious Anxiety Model. Those groups who are more likely to have developed MA as a result of negative academic experiences have a more profoundly lowered math performance relative to their absolute MA levels—possibly because the mechanisms of the Deficit Theory were what caused some or most of them to develop academic anxieties in the first place. We would, therefore, suggest that a reciprocal theory (see [7]) operates to explain the relationship between MA and math performance: MA can develop because of poor performance or in its absence, and lowers math performance to some degree regardless of why it develops. Thus those individuals who develop relatively high MA because of poor performance have lower performance than those who develop it simply because of anxiety predisposition.

### Implications for education and interventions

The finding that older students fall into varied anxiety profiles, and that these profiles affect their math performance above and beyond the effect of MA alone, highlights the importance of looking at the student as a whole rather than merely their MA levels. For example, when attempting to improve math performance in students with MA, different strategies could be developed depending on which anxiety profile a child falls into. Those who have MA as a result of a predisposition to all forms of anxiety may have lower math performance solely as a result of the interfering effects of anxiety. Thus an intervention targeted at reducing their anxiety levels could be of great benefit to their mathematics performance. On the other hand, those whose anxieties are specifically related to academia may have developed these anxieties as a direct result of negative experiences (including poor performance) in math. Acting directly to improve these children’s experiences and performance in math may be more likely to raise their performance than simply trying to reduce their levels of anxiety, and might have the knock-on effect of reducing their MA.

### Limitations and further study

The diagram in Fig 5 provides a cogent explanation of how different anxiety profiles could develop within individuals, which accords with our data. However, the mechanisms provided are vague and sparse, because this was a large-sample screening study rather than one which went into more detail as to the causes and nature of individual experiences of anxiety. In order to elaborate on the diagram in Fig 5, and possible explanations provided for the differing relationship between MA and performance depending on anxiety profile, it is necessary to conduct further cognitive testing and structured interviews with groups of students in the relevant anxiety profiles.

There may be specific academic factors which increase one’s risk of both test anxiety and MA. Whilst we have not measured these factors, suggestions for variables which might increase risk of anxiety about academic situations include low self-concept about school. This is linked with test anxiety and to poor math self-concept [39], which in turn is linked to MA [40,41]. Comparing groups of children with specific academic anxiety to those with academic anxieties as part of a “High anxiety” profile could help elucidate whether specific academic factors play more of a role in the former group than the latter.

For students with a predisposition towards general anxiety, it would be interesting to investigate what can be done to increase the chances of these students ending up with general anxiety only, rather than having high levels of all anxiety measured. For example, are there protective factors against developing academic anxiety, and if so what are these factors? This will involve comparing students in the “General anxiety” profile with those in the “High anxiety” profile.

### Conclusions

Our data suggest that the integration of person- and variable-centered analysis can lead to novel conclusions in the field of MA research. We note developmental changes in anxiety specificity between year 4 and year 7/8 students. In the older students (with more specific anxiety forms) it appears that students with high levels of anxiety generally suffer less of an MA-related performance decrement than those with specifically elevated academic anxieties. This could explain some of the huge variation in performance outcomes between individuals with a given level of MA. Investigating this further could lead to the development of targeted interventions, depending on a child’s profile of anxiety as a whole, rather than their MA alone.
